# Endothelial cells direct human mesenchymal stem cells for osteo- and chondro-lineage differentiation through endothelin-1 and AKT signaling

**DOI:** 10.1186/s13287-015-0065-6

**Published:** 2015-05-01

**Authors:** Tsung-Lin Tsai, Bowen Wang, Matthew W Squire, Lian-Wang Guo, Wan-Ju Li

**Affiliations:** Department of Orthopedics and Rehabilitation, Laboratory of Musculoskeletal Biology and Regenerative Medicine, University of Wisconsin-Madison, 1111 Highland Ave, WIMR 5051, Madison, WI 53705 USA; Department of Biomedical Engineering, University of Wisconsin-Madison, 1111 Highland Ave, WIMR 5051, Madison, WI 53705 USA; Department of Surgery, University of Wisconsin-Madison, 1111 Highland Ave, WIMR 5151, Madison, WI 53705 USA

## Abstract

**Introduction:**

Human mesenchymal stem cells (hMSCs) reside in a perivascular niche of the body, suggesting that they interact closely with vascular endothelial cells (ECs) through cell-cell interaction or paracrine signaling to maintain cell functions. Endothelin-1 (ET1) is a paracrine factor mainly secreted by ECs. We thus hypothesize that ECs can regulate cellular activities of hMSCs and direct their stem cell fate.

**Methods:**

We investigated whether co-cultured human aortic endothelial cells (HAECs) were able to regulate expression of potency- and lineage-related markers in bone marrow-derived hMSCs. We further explored the regulatory effects of ET1 on cell proliferation, expression of surface antigens and pluripotency-related markers, and multilineage differentiation in hMSCs. Activation of the AKT signaling pathway in hMSCs was also analyzed to identify its mechanistic role in the ET1-induced regulation.

**Results:**

Co-cultured HAECs enhanced expression of mesenchymal lineage-related markers in hMSCs. Treatment of ET receptor antagonist downregulated the increased expression of *CBFA1* in hMSCs cultured with HAEC-conditioned medium. hMSCs treated with ET1 showed cell proliferation and expression of surface antigens, CD73, CD90, and CD105, comparable with those without ET1 treatment. ET1-treated hMSCs also expressed upregulated mRNA transcript levels of *OCT3/4*, *NANOG*, *CBFA1* and *SOX9*. When induced for lineage-specific differentiation, hMSCs pre-treated with ET1 showed enhanced osteogenesis and chondrogenesis. However, adipogenic differentiation of hMSCs was not affected by ET1 pretreatment. We further showed that the ET1-induced regulation was mediated by activation of AKT signaling.

**Conclusion:**

Our results demonstrate that ET1 secreted by HAECs can direct bone marrow-derived hMSCs for osteo- and chondro-lineage differentiation through activation of the AKT signaling pathway, suggesting that ET1 plays a crucial role in regulation of hMSC activity. Our findings may help understand how hMSCs interact with ECs in a perivascular niche.

**Electronic supplementary material:**

The online version of this article (doi:10.1186/s13287-015-0065-6) contains supplementary material, which is available to authorized users.

## Introduction

Human mesenchymal stem cells (hMSCs) are multipotent and capable of self-renewal [1-3]. They can be isolated from various adult and fetal tissues [4-6], and can be induced into osteo-, chondro-, or adipo-lineage cells *in vitro* and *in vivo* [7]. With these features, hMSCs hold great potential for regenerative medicine applications. To explore the potential, extensive research effort has been devoted to understanding mesenchymal stem cell (MSC) biology and controlling MSC behavior.

While hMSCs regulated by physical or chemical signals have been studied in cell culture, the knowledge about hMSC behavior *in vivo*, particularly interaction with other cell types, remains largely unknown [8-10]. Recent studies have shown that properties of pericytes are similar to those of MSCs [11-14]. Researchers have also reported that hMSCs isolated from bone marrow express CD146, a surface marker of pericytes [15,16]. These reports concordantly indicate that hMSCs reside in a perivascular niche of the body. The anatomical relation between hMSCs and vascular endothelial cells (ECs) also suggests that these two cell types interact with each other likely through cell-cell interaction and/or paracrine signaling. Indeed, Kaigler and colleagues have demonstrated that ECs enhance osteogenic differentiation of hMSCs through direct cell-cell contact [17]. Using EC-conditioned medium for hMSC culture, Saleh and colleagues have shown that the paracrine signaling molecules secreted by ECs increase proliferation and osteogenic differentiation of hMSCs [18]. Another study by Saleh and colleagues has reported that ECs increase osteogenesis but decrease adipogenesis of hMSCs in a three-dimensional spheroid co-culture system [19]. Together these findings demonstrate that ECs are able to regulate hMSC activities. However, the underlying mechanism has not been fully identified.

Endothelin-1 (ET1) is a secretory factor produced by ECs [20,21]. Several research reports have demonstrated that ET1 is involved in the regulation of osteogenic differentiation, suggesting that ET1 may play a pivotal role in regulation of MSC activities. For example, von Schroeder and colleagues have shown that ET1 promotes osteoprogenitor proliferation and differentiation in fetal calvarial cell culture [22]. Clines and colleagues have demonstrated that ET signaling is an important regulator of postnatal trabecular bone formation [23,24]. In addition, a study by Salama and colleagues has shown that ET1 enhances proliferation and migration of hMSCs and increases expression of alpha smooth muscle actin in hMSCs [25]. These studies collectively suggest that ECs may secrete ET1 to regulate hMSC activities.

ET1 binds to endothelin receptor type A or B on the cell surface to activate downstream signaling molecules of several pathways, including mitogen activated-protein kinase [26,27], Ca^2+^/calmodulin-dependent protein kinase [28], protein kinase C [29,30], and phosphatidylinositol 3-kinase/AKT [31,32]. It is known that these signaling molecules play a role in regulation of MSC behavior. For example, our group has previously demonstrated that AKT signaling is involved in the regulation of hMSC senescence in hypoxic culture [33]. Inhibition of AKT signaling results in increased hMSC senescence and decreased expression of pluripotency-related markers. Other groups have also reported that activation of AKT signaling suppresses cell senescence, maintains stem cell properties, and regulates MSC differentiation [34-37]. Therefore, it is worthwhile to study whether ET1 regulates MSC activities through activation of AKT signaling.

In this study, we hypothesized that ECs secrete ET1 to regulate hMSC activities, and the regulatory effects of ET1 on hMSCs are mediated by the AKT signaling pathway. To test our hypothesis, bone marrow-derived hMSCs were co-cultured with human aortic endothelial cells (HAECs) or treated with recombinant ET1 in culture, and then analyzed for cell proliferation, expression of surface antigens, and multilineage differentiation. Activation of AKT signaling by ET1 in hMSCs was also analyzed to determine the underlying mechanism.

## Methods

### Human mesenchymal stem cell isolation and cell culture

Ethical approval of human tissue procurement for this study was granted by the Institutional Review Board at the University of Wisconsin-Madison. Bone marrow-derived hMSCs were harvested from femoral heads and necks of one male and two female patients undergoing total hip arthroplasty. The cells from each donor were individually cultured and assayed in this study. Approved by the Institutional Review Board, informed consent from patients was waived in accordance with federal regulations of human tissue obtained as surgical waste for biomedical research. hMSCs were isolated following a previously described protocol [38]. Briefly, after harvested from the interior compartment of femoral head and neck, bone marrow was mixed with Dulbecco’s modified Eagle medium (DMEM; Gibco, Carlsbad, CA, USA). A syringe with an 18-gauge needle was used to filter out bone debris from the bone marrow/DMEM mixture. The collected medium was then centrifuged at 1,000 rpm for 5 minutes. After removing the supernatant, the resulting cell pellet was reconstituted using 25 ml Hank's Balanced Salt Solution (Invitrogen, Carlsbad, CA, USA), and then slowly added into a 50 ml conical tube containing 20 ml Ficoll solution (GE Health, Pittsburgh, PA, USA). After centrifugation at 500 *g* for 30 minutes, mononuclear cells were collected and plated in cell culture flasks with culture medium composed of low-glucose DMEM, 10% fetal bovine serum (FBS; Atlanta Biologicals, Atlanta, GA, USA) and antibiotics. The cells were maintained in an incubator at 37°C in a humidified 5% CO_2_ atmosphere. When reaching 70 to 80% density confluence, the cells were trypsinized using 0.05% trypsin/EDTA (Gibco) and re-plated at a seeding density of 1,000 cells/cm^2^. Culture medium was replaced every 3 days. Cells between passages 2 and 4 were used in this study.

### Culture of human embryonic stem cell-derived mesenchymal stem cells

Human embryonic stem cell-derived (hESC)-MSCs were obtained from Dr. Igor Slukvin through collaboration. The cells were previously derived from H1 hESCs and thoroughly characterized [39]. The experiments involving hESC-MSCs were approved by the Institutional Biosafety Committee at the University of Wisconsin-Madison. After thawing, hESC-MSCs were plated in tissue culture plates coated with 5 μg/ml human fibronectin (Invitrogen) and 10 μg/ml human collagen type 1 (Stem Cell Technologies, Vancouver, Canada), and cultured in medium composed of 50% StemLine II hematopoietic stem cell serum-free medium (Sigma-Aldrich, St Louis, MO, USA), 50% Human Endothelial serum-free medium (Gibco), 100 μM monothioglycerol (Sigma-Aldrich), 1:100 dilution Glutamax (Gibco), 1:2,000 dilution ExCyte supplement (EMD Millipore, Billerica, MA, USA), 10 ng/ml fibroblast growth factor-2 (Peprotech, Rocky Hill, NJ, USA), and antibiotics. The cells were maintained in an incubator at 37°C in a humidified 5% CO_2_ atmosphere. When reaching 70 to 80% density confluence, the cells were collected using Accutase (Life Technologies, Carlsbad, CA, USA) and re-plated at a seeding density of 1,000 cells/cm^2^. Culture medium was replaced every 3 days.

### Co-culture of human mesenchymal stem cells and human aortic endothelial cells

HAECs derived from a female donor were obtained from Lonza (Lonza, Allendale, NJ, USA). After thawing, the cells were plated in tissue culture flasks with culture medium composed of Endothelial Basal Medium-2 (Lonza), 10% FBS and antibiotics, and maintained in an incubator at 37°C in a humidified 5% CO_2_ atmosphere. Cells between passages 5 and 7 were used for all experiments. When culture medium was replaced every 2 days, HAEC-conditioned medium was collected and stored in a −20°C freezer for later use.

**Figure 1:**
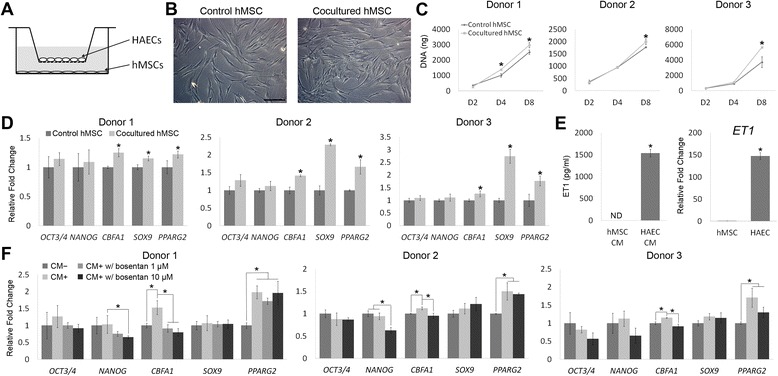
Activities of human mesenchymal stem cells (hMSCs) regulated by co-cultured human aortic endothelial cells (HAECs) or HAEC-conditioned medium. **(A)** Illustration of hMSC/HAEC Transwell co-culture setup. hMSCs were seeded at the bottom of wells while HAECs were seeded in Transwell inserts. **(B)** Micrographs of control hMSCs or hMSCs co-cultured with HAECs. Scale bar: 200 μm. **(C).** Proliferation of hMSCs co-cultured with or without HAECs was analyzed by quantifying total DNA content. **(D)** Relative mRNA expression levels of potency- and lineage-related markers in control hMSCs or hMSCs co-cultured with HAECs for 3 days were analyzed by quantitative RT-PCR. **(E)** Concentrations of soluble endothelin-1 (ET1) in hMSC- or HAEC-conditioned medium (CM) were quantified using enzyme-linked immunosorbent assay (left). Relative mRNA expression levels of *ET1* in hMSCs or HAECs were determined by quantitative RT-PCR (right). **(F)** Relative mRNA expression levels of potency- and lineage-related markers in hMSCs treated without HAEC-conditioned medium (CM-), or with HAEC-conditioned medium (CM+) added with or without bosentan for 3 days were analyzed by quantitative RT-PCR. *P < 0.05; n = 3. ND, not detectable.

To set up co-culture of hMSCs and HAECs in Transwell System (BD Biosciences, San Diego, CA, USA) as illustrated in Figure 1A, hMSCs were plated at the bottom of 6-well plates at a seeding density of 1,000 cells/cm^2^ and HAECs were plated in transwell inserts at a seeding density of 2,000 cells/cm^2^. The co-culture with medium composed of 50% hMSC culture medium and 50% HAEC culture medium was maintained at 37°C in a humidified 5% CO_2_ atmosphere.

To study the role of ET1 in regulation of hMSC activities, HAEC-conditioned medium was prepared by filtering the medium collected from HAEC culture using a 0.22 μm syringe filter (EMD Millipore) and mixing it with the same volume of fresh HAEC culture medium, and then using it to treat hMSCs with or without 1 or 10 μM bosentan (AvaChem Scientific, San Antonio, TX, USA), a non-selective endothelin receptor antagonist. hMSCs maintained with fresh HAEC culture medium served as a control.

### Cell proliferation analysis

hMSCs co-cultured with HAECs or treated with 0.01 or 0.1 μM ET1 in culture were harvested and digested overnight using Proteinase K (Sigma-Aldrich) to extract DNA. The total amount of double-stranded DNA was analyzed to determine cell proliferation using the PicoGreen assay (Invitrogen) following the manufacturer’s instructions.

Long-term growth of hMSCs in culture with or without 0.1 μM ET1 was determined by measuring accumulated population doublings (PDs). Cell number was calculated at each passage by counting the cells using a hemocytometer. The number of PDs was determined by the formula PD = log_2_ (C_1_/C_0_), where C_0_ = the number of cells initially seeded and C_1_ = the number of cells being passaged.

### Total RNA extraction and quantitative reverse transcription polymerase chain reaction

Total RNA was extracted from cells using the Nucleo Spin RNA II kit (Clontech, Mountain View, CA, USA) following the manufacturer’s instructions. Complementary DNA was transcribed from 500 ng RNA using the High-Capacity cDNA Reverse Transcription kit (Applied Biosystems, Carlsbad, CA, USA). Quantitative RT-PCR (qRT-PCR) was performed using the iQ SYBR Green Premix (BioRad, Hercules, CA, USA) with primers detecting *octamer binding transcription factor 3/4* (*OCT3/4*), *NANOG*, *core-binding factor subunit alpha-1* (*CBFA1*), *sex determining region Y-box 9* (*SOX9)*, *peroxisome proliferator-activated receptor gamma 2* (*PPARG2*), *ET1*, *alkaline phosphatase* (*ALP*), *osteocalcin* (*OC)*, *aggrecan* (*AGN*), *collagen type 2* (*COL2*), *lipoprotein lipase* (*LPL*) and *ubiquitin C* (*UBC*). OCT3/4 and NANOG are recognized pluripotent transcription factors in ESCs. As it has been reported that low levels of these factors is a feature of early passage hMSCs [40,41], the mRNA expression of *OCT3/4* and *NANOG* was analyzed to assess the undifferentiated state of hMSCs, and that of the other genes was analyzed to determine tissue lineage-specific differentiation [33]. The primer sequences are listed in Table 1. The expression level of each mRNA transcript was determined by referencing to that of *UBC* using the 2^-ΔCt^ method.

**Table 1:** **Primer sequences for quantitative RT-PCR analysis**

**Gene name**	**Accession number**	**Primer sequences (5′ to 3′)**
***OCT3/4***	NM_002701.4	F: TGGAGAAGGAGAAGCTGGAGCAAAA
		R: GGCAGATGGTCGTTTGGCTGAATA
***NANOG***	NM_021865.2	F: GCTGAGATGCCTCACACGGAG
		R: TCTGTTTCTTGACCGGGACCTTGTC
***CBFA1***	NM_004843.3	F: GGTTCCAGCAGGTAGCTGAG
		R: AGACACCAAACTCCACAGCC
***SOX9***	NM_000346.3	F: TAAAGGCAACTCGTACCCAA
		R: ATTCTCCATCATCCTCCACG
***PPARG2***	NM_138711.3	F: ATGACAGCGACTTGGCAATA
		R: GGCTTGTAGCAGGTTGTCTT
***ET1***	NM_001168319.1	F: AAGGCAACAGACCGTGAAAAT
		R: CGACCTGGTTTGTCTTAGGTG
***ALP***	NM_000478.3	F: CAAAGGCTTCTTCTTGCTGG
		R: GGTCAGAGTGTCTTCCGAGG
***OC***	NM_199173.3	F: GACTGTGACGAGTTGGCTGA
		R: GGAAGAGGAAAGAAGGGTGC
***AGN***	NM_013227.2	F: CACGATGCCTTTCACCACGAC
		R: TGCGGGTCAACAGTGCCTATC
***COL2***	NM_001844.4	F: CCTCTGCGACGACATAATCT
		R: CTCCTTTCTGTCCCTTTGGT
***LPL***	NM_000237.2	F: AGGAGCATTACCCAGTGTCC
		R: GGCTGTATCCCAAGAGATGGA
***UBC***	NM_021009.4	F: TGAAGACACTCACTGGCAAGACCA
		R: CAGCTGCTTTCCGGCAAAGATCAA

Forward and reverse primers are indicated as “F” and “R”, respectively.

### Quantification of endothelin-1 levels in culture

Conditioned medium was collected from hMSC or HAEC culture during medium changing and the collected medium from two lots of medium changing was combined for analysis. The concentration of soluble ET1 in hMSC- or HAEC-conditioned medium was determined using the ET1 enzyme-linked immunosorbent assay kit (R&D Systems, Minneapolis, MN, USA) following the manufacturer’s instructions.

### Flow cytometric analysis of mesenchymal stem cell surface antigens

hMSCs treated with or without 0.1 μM ET1 were trypsinized and washed twice using ice-cold phosphate-buffered saline containing 0.1% sodium azide and 1% bovine serum albumin (Sigma-Aldrich). The cells were then incubated with the antibodies against cell surface markers CD73, CD90 and CD105 (BD Biosciences) for 30 minutes at 4°C. After washing with the buffer three times to remove unbound antibodies, the cells were fixed with 1% paraformaldehyde solution, and then analyzed by flow cytometry (BD Biosciences). Data were analyzed using the FlowJo software (TreeStar, Ashland, OR, USA).

### Assessment of multilineage differentiation of human mesenchymal stem cells

To investigate whether ET1 can prime the capability of undifferentiated hMSCs for multilineage differentiation, hMSCs were maintained in culture with or without 0.1 μM ET1 for two passages and then induced for osteo-, adipo- and chondrogenesis without ET1 for 21 days. The cells were trypsinized and replated in tissue culture plates at a seeding density of 5,000 or 10,000 cells/cm^2^ for osteogenic or adipogenic differentiation, respectively. The cells were induced using osteogenic medium composed of low-glucose DMEM, 10% FBS, 10 mM β-glycerophosphate, 50 μg/ml L-ascorbic acid-2-phosphate, 0.1 μM dexamethasone (Sigma-Aldrich), and antibiotics, or using adipogenic medium composed of high-glucose DMEM, 10% FBS, 1 μM dexamethasone, 0.5 mM 3-isobutyl-1-methylxanthine, 1 μg/ml insulin (Sigma-Aldrich), and antibiotics. To induce hMSCs for chondrogenic differentiation, a previously described protocol with modifications was used [42]. Briefly, 250,000 cells were centrifuged in a 15-ml conical tube at 600 *g* for 5 minutes to form a high-density cell pellet. The cell pellet was induced using chondrogenic medium containing high-glucose DMEM, 1% ITS+ (BD Biosciences), 50 μg/ml L-ascorbic acid-2-phosphate, 0.1 μM dexamethasone, 40 μg/ml L-proline, 0.9 mM sodium pyruvate (Sigma-Aldrich), and antibiotics, supplemented with 10 ng/ml transforming growth factor beta-1 (TGFB1) (Peprotech). Differentiation medium was changed every 3 days during differentiation induction.

To analyze osteogenic differentiation of hMSCs, cells were fixed with 60% isopropanol after 21 days of induction. The cells were stained for Alizarin red (Rowley Biochemical, Danvers, MA, USA) to evaluate the extent of mineral deposition. To quantify the level of mineralization, calcium deposition in culture was extracted using 0.5 M hydrogen chloride, and then measured using the LiquiColor kit (Stanbio, Boerne, TX, USA) following the manufacturer’s protocol. For analyzing chondrogenic differentiation, after 21 days of induction, chondrogenic cell pellets were fixed with 4% formaldehyde solution, dehydrated using a series of concentrations of ethanol, infiltrated with xylene, and then embedded in paraffin. For histology analysis, the embedded cell pellets were cut into 8-μm sections using a microtome, deparaffinized, rehydrated, and then stained with Alcian blue (Polysciences, Warrington, PA, USA) to detect glycosaminoglycan (GAG). To quantify GAG production, chondrogenic cell pellets were digested with papain, and analyzed by the dimethylmethylene blue (DMMB) assay following a previously published protocol [43]. Briefly, 16 mg DMMB (Sigma-Aldrich) was dissolved in 1,000 ml water containing 3.04 g glycine, 1.6 g NaCl and 95 ml acetic acid. After 200 μl DMMB solution was mixed with 20 μl papain-digested sample solution, the mixture was measured for the absorbance at the wavelength of 525 nm to determine the GAG amount, which was then normalized with the DNA content determined by the PicoGreen assay. To evaluate adipogenic differentiation, hMSCs were fixed with 4% formaldehyde solution, and then stained with Oil Red O (Sigma-Aldrich) for lipid droplet formation after 21 days of induction. After image analysis, the staining of Oil Red O in culture was dissolved by 2-propanol. The Oil Red O solution was then analyzed for the absorbance at the wavelength of 656 nm to determine the amount of lipid droplets.

### Protein extraction and western blotting analysis

To extract protein from hMSCs, the cells were lyzed using RIPA buffer composed of 50 mM Tris–HCl (pH 7.5), 0.25% Na-deoxycholate, 1% Nonidet P-40, 150 mM NaCl, 1 mM EDTA, and complete protease inhibitor cocktail (Roche, Indianapolis, IN, USA). After centrifugation at 14,000 rpm for 10 minutes, the supernatant was collected. Protein concentration was measured using the BCA Protein Assay kit (Pierce, Rockford, IL, USA). A 40-μg protein sample was loaded into each lane of a 10% polyacramide gel (Bio-Rad) for electrophoresis, and the separated proteins were then transferred from the gel onto a polyvinylidene fluoride membrane (Bio-Rad). The membrane was incubated with primary antibodies against AKT, phospho-AKT (Ser473), and glyceraldehyde 3-phosphate dehydrogenase (Cell Signaling, Danvers, MA, USA) in a blocking solution composed of Tris-buffered saline containing 5% nonfat milk (Bio-Rad) and 0.1% Tween 20 (Sigma-Aldrich) overnight at 4°C. After removing unbound antibodies, the membrane was incubated with horseradish peroxidase-linked secondary antibody (Cell Signaling) in the blocking solution for 1 hour at room temperature. The immuno-detected protein bands on the membrane were visualized using the SuperSignal West Pico Chemiluminescent Substrate (Pierce), and then documented by the Kodak Image Station 4000R Pro system (Kodak, Rochester, NY, USA).

### Regulation of AKT signaling

To regulate the activation of the AKT signaling pathway, 0.01 or 0.1 μM AKT Inhibitor IV (EMD Millipore) was used in hMSC culture. Specifically, ET1-treated hMSCs were cultured with or without AKT Inhibitor IV for two passages before qRT-PCR analysis or induction for multilineage differentiation.

### Statistical analysis

All quantitative data of assays that analyze three donors’ cells were presented as the mean ± standard deviation, as the assays were performed with samples in technical triplicate (n = 3). A Student’s *t*-test or one-way analysis of variance with *post-hoc* Tukey’s test was used for statistical comparison. A *P*-value <0.05 was considered statistically significant.

## Results

### Human aortic endothelial cells secreted endothelin-1 to regulate human mesenchymal stem cell activities

We first used hMSC/HAEC co-culture to investigate the effects of paracrine factors on hMSCs (Figure 1A) and hMSCs without HAEC as control culture. After 3 days of culture, cell morphologies of control and co-cultured hMSCs were similar (Figure 1B). The cell number of hMSCs co-cultured with HAECs was higher than that of control hMSCs at day 8 (Figure 1C), suggesting that HAECs are able to increase proliferation of hMSCs. qRT-PCR analysis of potency- and lineage-related markers showed that among cells from all three donors, hMSCs in co-culture expressed higher mRNA levels of lineage-related transcription factors *CBFA1*, *SOX9*, and *PPARG2* than control hMSCs, while the expression levels of *OCT3/4* and *NANOG* were comparable between co-cultured and control hMSCs (Figure 1D). To investigate whether ET1 secreted by HAECs in co-culture is able to regulate hMSCs, we first quantified levels of ET1 in hMSC- and HAEC-conditioned medium. The ET1 level in HAEC-conditioned medium was 1,537 ± 86 pg/ml whereas that in hMSC-conditioned medium was not detectable (Figure 1E, left). Similarly, the mRNA expression level of *ET1* in HAECs was about 147-fold higher than that in hMSCs (Figure 1E, right). We next used bosentan, a non-selective endothelin receptor antagonist, to inhibit ET1 signaling in hMSCs. Considering the possibility that adding bosentan directly in hMSC/HAEC co-culture may affect HAECs and subsequently interfere with hMSC response, we decided to treat hMSCs with bosentan in HAEC-conditioned medium instead of in Transwell co-culture. The mRNA expression levels of *CBFA1* and *PPARG2* of donor 1 hMSCs cultured with HAEC-conditioned medium were higher than those of control hMSCs cultured without conditioned medium, while the levels of *OCT3/4*, *NANOG*, and *SOX9* were comparable between the two cultures (Figure 1F). Compared to hMSCs cultured in HAEC-conditioned medium without bosentan, the cells in conditioned medium with 10 μM bosentan expressed a decreased level of *NANOG* and those cultured with either 1 or 10 μM bosentan showed downregulated expression of *CBFA1* (Figure 1F). Similar to the results of donor 1 cells, donors 2 and 3 hMSCs cultured in conditioned medium treated with 10 μM bosentan showed consistent result patterns compared to their control hMSCs. These results indicate that inhibition of ET1 signaling attenuates the effects of HAEC-conditioned medium on regulation of hMSC activities.

### Endothelin-1 upregulated potency- and lineage-related markers in pre-differentiated human mesenchymal stem cells

**Figure 2:**
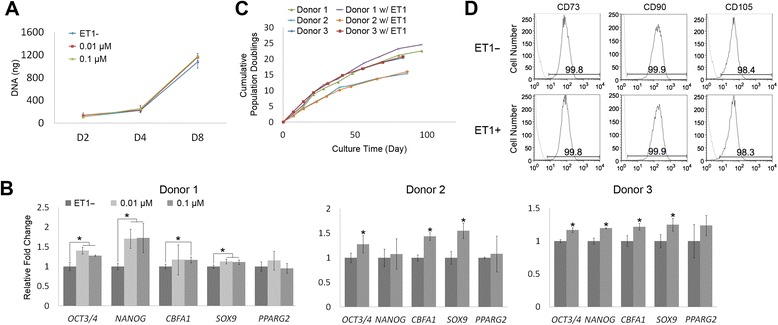
Effects of recombinant endothelin-1 (ET1) on regulation of pre-differentiated human mesenchymal stem cells (hMSCs). **(A)** Proliferation of hMSCs treated with or without different doses of ET1 was analyzed by quantifying total DNA content. **(B)** Relative mRNA expression levels of potency- and lineage-related markers in hMSCs treated with or without different doses of ET1 for two passages were analyzed by quantitative RT-PCR. *P < 0.05; n = 3. **(C)** Growth curves of hMSCs treated with or without 0.1 μM ET1 were generated by calculating cumulative population doublings. **(D)** Expression of surface antigens of hMSCs treated with or without 0.1 μM ET1 for two passages was assessed by flow cytometry. Area under black curve: cells labeled with target antibody; area under gray curve: cells labeled with isotype antibody.

To study the effects of ET1 on hMSC activities, we next treated hMSCs with recombinant ET1 in culture. Cell numbers at days 2, 4 or 8 were comparable between culture treated with or without ET1 (Figure 2A), suggesting that ET1 does not affect proliferation of hMSCs. We further analyzed mRNA expression of potency- and lineage-related markers of hMSCs under the effect of ET1. The results showed that the mRNA levels of *OCT3/4* and *NANOG* in donor 1 hMSCs treated with ET1 were higher than those in control hMSCs without ET1 (Figure 2B). In addition, compared to the cells without ET1 treatment, hMSCs treated with 0.1 μM ET1 expressed increased levels of *CBFA1* and *SOX9* while those treated with 0.01 μM ET1 expressed only increased *SOX9*. Donor 2 cells showed that the expression levels of *OCT/4*, *CBFA1* and *SOX9* in culture treated with 0.1 μM ET1 were higher than those in culture without ET1. The results of donor 3 cells were similar to those of donor 1 cells or those of donor 2 cells except for the expression level of *NANOG*. The expression levels of *PPARG2* were comparable among all cultures regardless of donor source. These findings suggest that ET1 enhances the potency of hMSCs and directs the cells toward the osteogenic or chondrogenic lineage, and the response of hMSCs is not ET1 dose-dependent. We thus decided to use the concentration of 0.1 μM to treat cells in the subsequent experiments. The results of long-term cell growth showed that the cumulative PDs of hMSCs treated with or without ET1 were comparable (Figure 2C), suggesting that ET1 does not affect growth of hMSCs in culture. Lastly, the expression of surface antigens of hMSCs analyzed by flow cytometry showed that the cell population treated with ET1 expressed the levels of CD73, CD90 or CD105 comparable with that in the cell population without ET1 treatment (Figure 2D). These findings demonstrate that ET1 treatment does not affect expression of hMSC surface markers.

### Endothelin-1 enhanced osteogenic and chondrogenic capacities of pre-differentiated human mesenchymal stem cells

**Figure 3:**
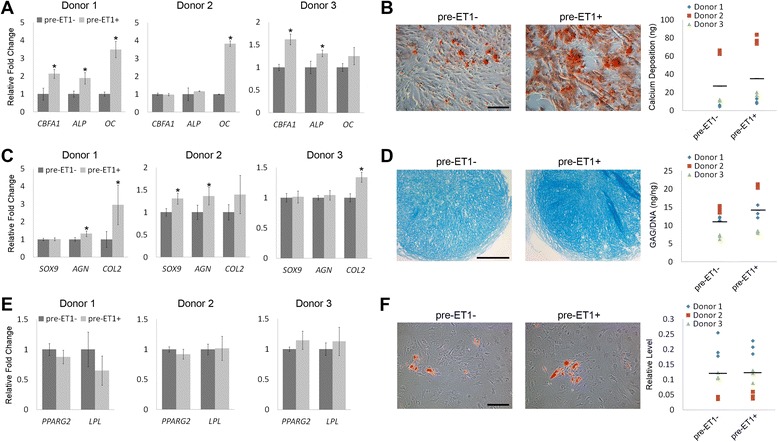
Effects of endothelin-1 (ET1) on regulation of lineage-specific differentiation capacity of pre-differentiated human mesenchymal stem cells (hMSCs). hMSCs pretreated with (pre-ET1+) or without ET1 (pre-ET1-) for two passages were induced for osteogenesis **(A,B)**, chondrogenesis **(C,D)**, or adipogenesis **(E,F)** for 21 days. (A,B) Osteogenesis was evaluated by analyzing relative mRNA expression levels of bone-related markers (A) or Alizarin red staining and quantification of calcium deposition (B). (C,D) Chondrogenesis was assessed by analyzing relative mRNA expression levels of cartilage-related markers (C) or Alcian blue staining and quantification of glycosaminoglycan (GAG) production (D). (E,F) Adipogenesis was evaluated by analyzing relative mRNA levels of fat-related markers (E) or Oil Red O staining and quantification of lipid droplets (F). *P < 0.05; n = 3. Scale bar: 200 μm.

We next investigated whether the upregulated expression of *CBFA1* and *SOX9* in ET1-treated hMSCs were able to drive enhanced osteogenesis and chondrogenesis upon differentiation induction. To this end, hMSCs cultured with or without ET1 for two passages were induced into osteogenic, chondrogenic, or adipogenic lineage using lineage-specific differentiation medium without ET1. After 21 days of osteogenesis, the mRNA levels of bone-related *CBFA1*, *ALP* and *OC* were upregulated in ET1-pretreated donor 1 hMSCs, compared to those in hMSCs without ET1 pretreatment (Figure 3A). ET1-pretreated donor 2 cells showed increased levels of *OC* and ET1-pretreated donor 3 cells expressed upregulated levels of *CBFA1* and *ALP* compared to their control hMSCs without ET1 pretreatment (Figure 3A). In addition to increased mRNA expression of bone-related markers, ET1-pretreated hMSCs showed greater intensity of Alizarin red staining than those without ET1 pretreatment (Figure 3B, left) and all three donors’ hMSCs pretreated with ET1 were able to produce more calcium deposition than their control cells (Figure 3B, right). The results of qRT-PCR and calcium deposition analyses indicate that ET1 increases the osteogenic capacity of pre-differentiated hMSCs. To investigate the effect of ET1 on the chondrogenic capacity of pre-differentiated hMSCs, the cells pretreated with or without ET1 were made into high-density cell pellets and induced for chondrogenesis for 21 days. Cell pellets made of ET1-pretreated donor 1 hMSCs showed higher mRNA levels of cartilage-related *AGN* and *COL2* than those made of hMSCs without ET1 pretreatment (Figure 3C). Cell pellets made of ET1-pretreated donor 2 or 3 hMSCs expressed upregulated mRNA levels of *SOX9* and *AGN* or increased levels of *COL2*, respectively, compared to its control cells without ET1 pretreatment (Figure 3C). Histological analysis showed greater intensity of Alcian blue staining in cell pellets of ET1-pretreated hMSCs than that in cell pellets of hMSCs without ET1 pretreatment (Figure 3D, left). Moreover, cell pellets of all three donors’ hMSCs pretreated with ET1 produced more GAGs than those of their control hMSCs without being pretreated with ET1 (Figure 3D, right). These findings suggest that ET1 upregulates the chondrogenic capacity of pre-differentiated hMSCs. Adipogenic differentiation of ET1-pretreated hMSCs was also examined. After 21 days of induction, hMSCs from all three donors showed comparable mRNA levels of *PPARG2* and *LPL* between cultures with or without ET1 pretreatment (Figure 3E). Analysis of lipid droplet production detected by Oil Red O showed similar intensity of staining (Figure 3F, left) and comparable amounts of lipid droplets in hMSCs pretreated with or without ET1 (Figure 3F, right), suggesting that ET1 does not affect the adipogenic capacity of pre-differentiated hMSCs. These results, together with those shown in Figure 2B, suggest that ET1 primes hMSCs for osteogenic and chondrogenic differentiation by upregulating the expression of *CBFA1* and *SOX9*.

### Endothelin-1 regulated human mesenchymal stem cell activities through activation of AKT signaling

**Figure 4:**
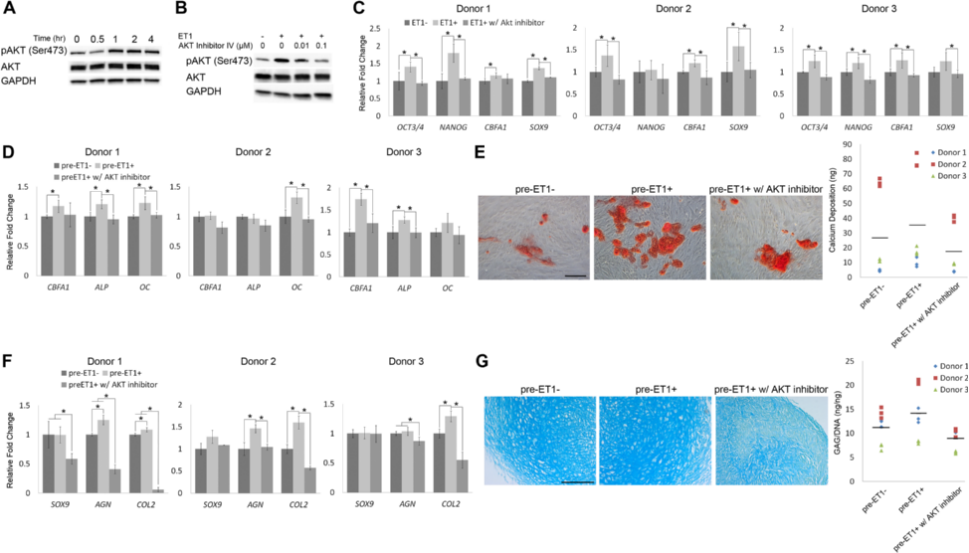
Response of endothelin-1 (ET1)-treated human mesenchymal stem cells (hMSCs) to regulation of AKT activity. **(A)** Western blotting analysis of AKT activation in hMSCs after ET1 induction. **(B)** Western blotting analysis of AKT activity in hMSCs 2 hours after ET1 induction. The cells were treated with or without AKT Inhibitor IV before ET1 induction. **(C)** Relative mRNA expression levels of potency- and lineage-related markers in ET1-treated hMSCs with or without AKT inhibition for two passages were analyzed by quantitative RT-PCR. **(D,E)** hMSCs pretreated with or without ET1 or AKT inhibitor for two passages were induced for 21-day osteogenesis. Osteogenesis was evaluated by analyzing relative mRNA expression levels of bone-related markers (D) or Alizarin red staining and quantification of calcium deposition (E). **(F,G)** hMSCs pretreated with or without ET1 or AKT inhibitor for two passages were made into cell pellets and induced for 21-day chondrogenesis. Chondrogenesis was assessed by analyzing relative mRNA expression levels of cartilage-related markers (F) or Alcian blue staining and quantification of glycosaminoglycan (GAG) production (G). *P < 0.05; n = 3. Scale bar: 200 μm.

To test our hypothesis that ET1 regulates hMSC activities through activation of the AKT signaling pathway, we first analyzed AKT activation upon ET1 induction in hMSCs. The results of western blotting showed that AKT in hMSCs was activated 1, 2, or 4 hours after ET1 induction (Figure 4A). To attenuate AKT activity, we used AKT Inhibitor IV in ET1-treated hMSCs. Among the ET1-treated cells, western blotting analysis showed that the level of phospho-AKT with 0.01 or 0.1 μM AKT Inhibitor IV treatment was decreased compared to that without being treated with AKT inhibitor (Figure 4B). Based on this result, we decided to use 0.1 μM AKT Inhibitor IV to effectively attenuate AKT activity in the subsequent experiments. We then examined the expression of potency- and lineage-related markers in ET1-treated hMSCs with or without AKT inhibition. We found that the mRNA levels of *OCT3/4*, *NANOG*, and *SOX9* in ET1-treated donor 1 hMSCs with AKT inhibitor were downregulated compared to those without AKT inhibitor, while the levels of *CBFA1* were comparable between the culture with and without AKT inhibitor (Figure 4C). The mRNA levels of *OCT3/4*, *CBFA1* and *SOX9* in ET1-treated donor 2 hMSCs and those of *OCT3/4*, *NANOG*, *CBFA1* and *SOX9* in ET1-treated donor 3 hMSCs treated with AKT inhibitor were decreased compared to those of their control cells without being attenuated by AKT inhibitor (Figure 4C). Moreover, after being treated with or without ET1 or AKT Inhibitor IV for two passages, the cells were then induced for osteogenesis and chondrogenesis without ET1 or AKT inhibitor. The results showed that after 21 days of osteogenic induction, donor 1 hMSCs pretreated with ET1 and AKT inhibitor expressed lower mRNA levels of *ALP* and *OC* than those with ET1 but without AKT inhibitor (Figure 4D). Donor 2 or 3 hMSCs pretreated with ET1 and AKT inhibitor expressed lower levels of *OC* or *CBFA1* and *ALP*, respectively, than those of its control cells with ET1 but without AKT inhibitor (Figure 4D). Analysis of Alizarin red staining showed decreased staining intensity in ET1- and AKT inhibitor-pretreated hMSCs, compared to that in ET1-pretreated cells (Figure 4E, left). The amount of calcium deposition produced by ET1- and AKT inhibitor-pretreated hMSCs was also less than that of ET1-pretreated cells (Figure 4E, right). For the analysis of chondrogenesis, after 21 days of induction, cell pellets made of ET1- and AKT inhibitor-pretreated donor 1 hMSCs expressed lower mRNA levels of *SOX9*, *AGN* and *COL2* than those made of ET1-pretreated hMSCs or control hMSCs without ET1 pretreatment (Figure 4F). Cell pellets made of ET1- and AKT inhibitor-pretreated donor 2 or 3 hMSCs expressed lower levels of *AGN* and *COL2* than those made of ET1-pretreated hMSCs (Figure 4F). Moreover, intensity of Alcian blue staining in cell pellets of ET1- and AKT inhibitor-pretreated hMSCs was decreased, compared to that in cell pellets of ET1-pretreated hMSCs or control hMSCs (Figure 4G, left). The GAG content in cell pellets made of ET1- and AKT inhibitor-pretreated hMSCs of all three donors was also lower than that in cell pellets made of ET1-pretreated cells (Figure 4G, right). Together these results showed that ET1 activated AKT signaling in hMSCs, and inhibiting AKT activity attenuated the effects of ET1 on upregulation of the osteogenic and chondrogenic capacities of pre-differentiated hMSCs.

### Endothelin-1 increased expression of human mesenchymal stem cell surface markers and osteogenic capacity of human embryonic stem cell-derived mesenchymal stem cells

We next examined the effect of ET1 on hESC-MSCs, another type of hMSC, to determine whether the regulation by ET1 is specific to bone marrow-derived MSCs or general to a variety of hMSCs regardless of their sources. The expression of potency- and lineage-related markers showed that the mRNA level of *CBFA1* in hESC-MSCs treated with ET1 was significantly higher than that in hESC-MSCs without ET1 treatment, while the levels of *OCT3/4*, *NANOG*, and *SOX9* were comparable between cells treated with and without ET1 (Additional file 1: Figure S1A), suggesting that ET1 directs hESC-MSCs toward the osteogenic lineage. Moreover, we found that after ET1 treatment in the cell population of hESC-MSCs treated with ET1, the percentages of hESC-MSCs expressing CD73, CD90, or CD105 were increased from 91.9%, 57.6%, or 56.7% to 94.8%, 78.8%, or 79.9%, respectively (Additional file 1: Figure S1B). This finding suggests that ET1 treatment may select a subpopulation of hESC-MSCs or prime the cells toward the mesenchymal lineage. hESC-MSCs treated with ET1 for two passages were then induced without ET1 for osteogenesis or chondrogenesis. After 21 days of osteogenesis, the mRNA levels of bone-related *CBFA1* and *ALP* were upregulated in ET1-pretreated hESC-MSCs, compared to those in hESC-MSCs without ET1 pretreatment (Additional file 1: Figure S1C). Analysis of mineral deposition using Alizarin red staining showed mineralization in ET1-pretreated hESC-MSC culture, while intensity of the staining was barely detectable in hESC-MSC culture without ET1 pretreatment (Additional file 1: Figure S1D, left). The amount of calcium deposition produced by ET1-pretreated hESC-MSCs was also significantly more than that produced by control hESC-MSCs without ET1 pretreatment (Additional file 1: Figure S1D, right). These results indicate that ET1 enhances the osteogenic capacity of pre-differentiated hESC-MSCs. For the effect of ET1 pretreatment on hESC-MSC chondrogenesis, cell pellets made of hESC-MSCs pretreated with or without ET1 induced for 21 days expressed comparable mRNA levels of *SOX9*, *AGN* and *COL2* (Additional file 1: Figure S1E). Alcian blue staining showed similar levels of intensity (Additional file 1: Figure S1F, left) and DMMB analysis indicated comparable amounts of GAG production (Additional file 1: Figure S1F, right) between chondrogenic cell pellets made of hESC-MSCs pretreated with and without ET1, suggesting that ET1 does not affect the chondrogenic capacity of pre-differentiated hESC-MSCs.

## Discussion

**Figure 5:**
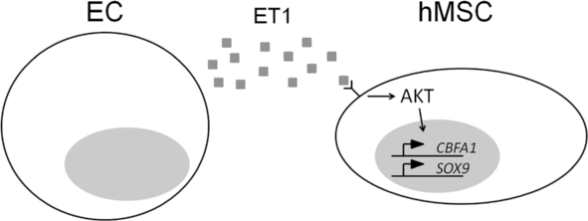
A proposed mechanistic model to illustrate the interaction between endothelial cells (ECs) and human mesenchymal stem cells (hMSCs). Endothelin-1 (ET1) secreted by ECs is able to activate AKT signaling in hMSCs to upregulate *CBFA1* and *SOX9*, thereby increasing the osteogenic and chondrogenic capacities.

In this study, we demonstrate that co-cultured ECs secrete ET1 to upregulate the osteogenic and chondrogenic capacities of pre-differentiated hMSCs. We further demonstrate that the effects of ET1 on hMSCs are mediated by AKT signaling. Based on our findings, we propose a working model describing the mechanism by which ECs regulate the osteogenic and chondrogenic capacities of pre-differentiated hMSCs through secreted ET1 activating AKT signaling (Figure 5).

hMSCs residing in a perivascular niche lie in close proximity to ECs [11,14]. Our study uses hMSC/HAEC co-culture, HAEC-conditioned medium, and ET1-treated culture to investigate activities of bone marrow-derived hMSCs regulated by ECs and soluble ET1. Specifically, pre-differentiated hMSCs co-cultured with HAECs or treated with ET1 were shown to increase expression of *CBFA1* and *SOX9*, indicating that ECs and their secreted ET1 play an important role in regulation of hMSC osteogenesis and chondrogenesis. Unlike the hematopoietic stem cell niche that has been extensively studied, the biological regulation in the hMSC niche remains largely unknown [44,45]. Our results provide insight into the regulatory mechanism underlying the interaction between hMSCs and ECs, which may help gain a better understanding of hMSC behavior in a perivascular niche.

Our results show that behavior of hMSCs co-cultured with HAECs is different from that of hMSCs treated with recombinant ET1. hMSCs treated with recombinant ET1 express increased levels of *OCT3/4* and *NANOG* while those co-cultured with HAECs show comparable expression levels of these two mRNA transcripts, compared to their control cells. In addition, the proliferation results indicate that co-cultured HAECs enhance hMSC propagation but ET1 does not affect proliferation of hMSCs. The discrepancy between hMSC activities regulated by ET1 and co-cultured HAECs suggests that ET1 is not the only soluble factor secreted by HAECs contributing to the results of hMSC regulation demonstrated in this study. Other molecules, such as platelet-derived growth factor, fibroblast growth factor, Wnt, bone morphogenetic protein, and Notch, have also been reported to be involved in the regulation of hMSC activities by ECs [18,19,46]. Further, our results also show that the expression of *SOX9* is enhanced in hMSCs co-cultured with HAECs but not in the cells cultured with HAEC-conditioned medium. This is probably due to different extents of ET1 stimulation associated with our culture setup: hMSCs with co-cultured HAEC were stimulated by continually released ET1 whereas those with HAEC-conditioned medium only by a single dose of ET1. Nevertheless, we believe that our results analyzed based on three donors’ cells collectively suggest that ET1 pretreatment can enhance the osteogenic and chondrogenic capacities of pre-differentiated hMSCs.

In this study, we investigate the effect of ET1 on hMSCs prior to differentiation and demonstrate a new finding that ET1 enhances the osteogenic capacity of pre-differentiated hMSCs through upregulation of *CBFA1*. Previous studies have also demonstrated that ET1 is able to enhance osteogenesis [22-24], but unlike our experimental setup focusing on the pre-differentiation stage, they study the effect of ET1 on osteogenesis during differentiation induction. These findings together may suggest that ET1 is able to regulate osteogenesis of hMSCs either prior to or during osteogenic induction through different mechanisms. In addition, our results show that ET1 does not affect proliferation of hMSCs, which is not in agreement with the previous finding suggesting that ET1 promotes proliferation of hMSCs [25]. The discrepancy between their and our findings in cell proliferation regulated by ET1 may be attributed to the difference in hMSC property. hMSCs used in our study are isolated from bone marrow whereas the cells used in their study are harvested from lung. It is possible that bone marrow- and lung-derived hMSCs have different biological properties that may prompt different degrees of cell proliferation in response to ET1 stimulation. While seeming to be rational, this hypothesis needs to be tested by a further study.

Our results show that ET1 induces activation of AKT signaling in pre-differentiated hMSCs to upregulate their osteogenic and chondrogenic capacities. Specifically, we demonstrate that activation of AKT signaling by ET1 upregulates expression of *CBFA1* and *SOX9* in hMSCs, which primes the cells for osteogenic and chondrogenic induction. Previous studies have shown that the AKT signaling pathway is associated with the regulation of *CBFA1* or *SOX9* in other cell types. For example, Ling and colleagues have reported that activation of AKT signaling upregulates the expression of *CBFA1* to enhance osteogenesis of MC3T3-E1 pre-osteoblast cells [47]. The study by Cheng and colleagues or Ikegami and colleagues has shown that the expression of *SOX9* is regulated by the AKT signaling pathway in nucleus pulposus cells [48] or chondrocytes [49], respectively. In this study, we demonstrate that attenuation of AKT signaling in pre-differentiated hMSCs inhibits chondrogenic differentiation. A previous report by Li and colleagues has shown that TGFB induces activation of AKT in precartilaginous stem cells, and inhibition of AKT activity suppresses TGFB-induced expression of *SOX9*, *AGN*, and *COL2* [50], indicating the crucial role of AKT signaling in chondrogenesis. In addition to the AKT signaling pathway, other pathways, such as the mitogen activated-protein kinase pathway [26,27] or the Ca^2+^/calmodulin-dependent protein kinase cascade [28], activated by ET1, may also be involved in the regulation of hMSC activities induced by ECs. However, investigation into how ECs regulate hMSCs through other signaling pathways is beyond the scope of this study. We plan to carry out the investigation in future studies.

hMSCs isolated or derived from adult tissue and embryonic sources are likely to have different cell properties and behavior [51]. For example, studies have shown that compared to human bone marrow-derived MSCs, hMSCs derived from hESCs are less inducible for mesenchymal lineage-specific differentiation [52,53]. Our results demonstrate that ET1 is able to increase the osteogenic capacity of hESC-MSCs by upregulating the expression of *CBFA1*, suggesting that ET1 primes pre-differentiated hESC-MSCs for subsequent osteogenic induction. Considering our finding that both human bone marrow- and ESC-derived MSCs are primed by ET1 for osteogenic induction, we hereby hypothesize that the effect of ET1 on priming the osteogenic capacity of hMSCs is ubiquitous among the cells derived from different sources. On the other hand, ET1 does not enhance the expression of *SOX9* in pre-differentiated hESC-MSCs nor chondrogenesis as it does in bone marrow-derived hMSCs, suggesting that the effect of ET1 on regulation of pre-differentiated hESC-MSCs for chondrogenesis and for osteogenesis may be different. Interestingly, our flow cytometry results seem to suggest that hESC-MSCs are composed of heterogeneous cell populations [53,54]. We demonstrate that ET1 increases the percentage of the cells expressing hMSC-related surface markers in a hESC-MSC population. In addition to our findings, previous studies have shown that ET1 is able to promote epithelial-to-mesenchymal [55,56] or endothelial-to-mesenchymal transition [57] in various cell types through the ET1/endothelin receptor type A signaling pathway. Taken together, our study demonstrates that ET1 plays an important role not only in regulation of biological response of adult tissue-derived MSCs but also in directing hESCs into MSC-like cells.

## Conclusions

It will enhance our knowledge of how hMSCs behave and function in a perivascular niche if we better understand the interaction between hMSCs and ECs. The knowledge is important to developing potential applications in tissue engineering and regenerative medicine. Our results demonstrate that ECs can secrete ET1 to regulate pre-differentiated hMSCs for subsequent induction of osteogenic and chondrogenic differentiation, and the regulation is mediated through the AKT signaling pathway. Our findings provide insight into one of the mechanisms governing how ECs regulate hMSC activities.

## Note

This article is part of an ‘Emerging Investigators’ collection showcasing the work of early career investigators who have demonstrated growing leadership in the field of stem cells and regenerative medicine. Other articles in the series can be found online at http://stemcellres.com/series/emerginginvestigators.

## Box 1. About Wan-Ju Li

**WJL** is an Assistant Professor in the Departments of Orthopedics and Rehabilitation, and Biomedical Engineering at the University of Wisconsin-Madison. He is also a faculty member in the Stem Cell and Regenerative Medicine Center. He leads the Musculoskeletal Biology and Regenerative Medicine Laboratory. WJL received a MS in Biomedical Engineering from Drexel University, a PhD in Cell and Tissue Engineering from Thomas Jefferson University, and postdoctoral training in mesenchymal stem cell biology at the National Institutes of Health. He was the recipient of the NASS Young Investigator Research Award and 3 M Faculty Award. His research interests include mesenchymal stem cell biology, musculoskeletal tissue engineering, and orthopedic regenerative medicine, with emphasis on understanding the effect of environmental factors on mesenchymal stem cells in bone marrow niches and developing viable approaches to differentiate mesenchymal stem cells into connective tissue lineage-specific cells in a controlled manner for regenerative applications.

## Additional file

Additional file 1: Figure S1. Effects of ET1 on regulation of hESC-MSC activities. **(A)** Relative mRNA expression levels of potency- and lineage-related markers in hESC-MSCs treated with or without 0.1 μM ET1 for two passages were analyzed by qRT-PCR. **(B)** Expression of surface antigens of hESC-MSCs treated with or without 0.1 μM ET1 for two passages was assessed by flow cytometry. Area under black curve: cells labeled with target antibody; area under gray curve: cells labeled with isotype antibody. **(C,D)** hESC-MSCs pretreated with or without ET1 were induced for 21-day osteogenesis. Osteogenesis was assessed by analyzing relative mRNA expression levels of bone-related markers (C) or Alizarin red staining and quantification of calcium deposition (D). Scale bar: 200 μm. **(E,F)** Pellets made of hESC-MSCs pretreated with or without ET1 were induced for 21-day chondrogenesis. Chondrogenesis was evaluated by relative mRNA expression levels of cartilage-related markers (E) or Alcian blue staining and quantification of GAG production (F). Scale bar: 100 μm. **P* < 0.05; n = 3.

## Abbreviations

DMEM: Dulbecco’s modified Eagle medium
DMMB: dimethylmethylene blue
EC: endothelial cell
ET1: endothelin-1
FBS: fetal bovine serum
GAG: glycosaminoglycan
HAEC: human aortic endothelial cell
hESC: human embryonic stem cell
hMSC: human mesenchymal stem cell
MSC: mesenchymal stem cell
PD: population doubling
qRT-PCR: quantitative reverse transcription polymerase chain reaction
TGFB1: transforming growth factor beta-1
