# Supplementary figures and images for: Endothelial cells direct human mesenchymal stem cells for osteo- and chondro-lineage differentiation through endothelin-1 and AKT signaling
Source: Stem Cell Res Ther. 2015 May 1;6(1):88. doi: 10.1186/s13287-015-0065-6 (PMC4416238; doi:10.1186/s13287-015-0065-6)

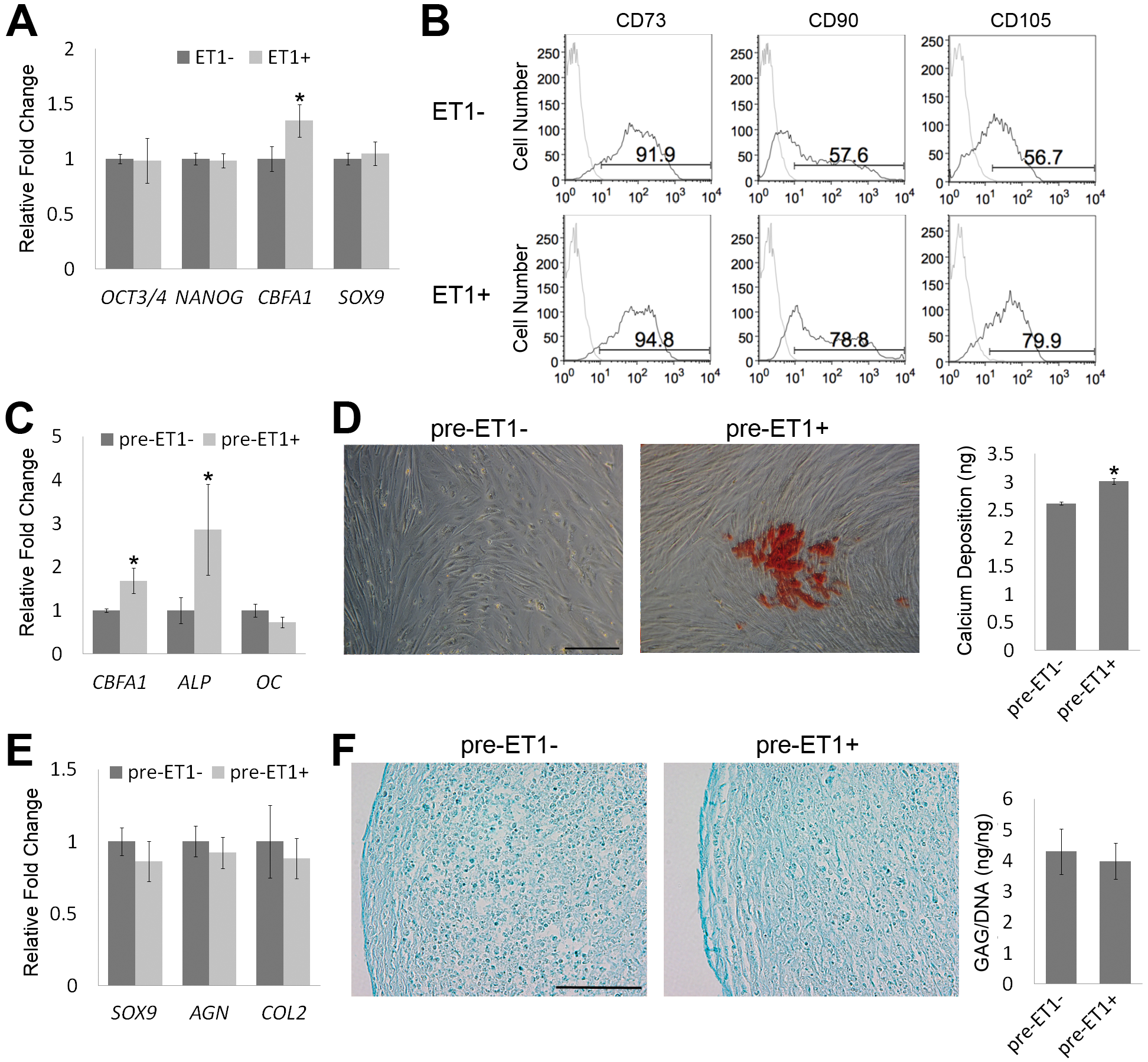

Supplement: Additional file 1: Figure S1. — Effects of ET1 on regulation of hESC-MSC activities. (A) Relative mRNA expression levels of potency- and lineage-related markers in hESC-MSCs treated with or without 0.1 μM ET1 for two passages were analyzed by qRT-PCR. (B) Expression of surface antigens of hESC-MSCs treated with or without 0.1 μM ET1 for two passages was assessed by flow cytometry. Area under black curve: cells labeled with target antibody; area under gray curve: cells labeled with isotype antibody. (C,D) hESC-MSCs pretreated with or without ET1 were induced for 21-day osteogenesis. Osteogenesis was assessed by analyzing relative mRNA expression levels of bone-related markers (C) or Alizarin red staining and quantification of calcium deposition (D). Scale bar: 200 μm. (E,F) Pellets made of hESC-MSCs pretreated with or without ET1 were induced for 21-day chondrogenesis. Chondrogenesis was evaluated by relative mRNA expression levels of cartilage-related markers (E) or Alcian blue staining and quantification of GAG production (F). Scale bar: 100 μm. *P < 0.05; n = 3. [file 13287_2015_65_MOESM1_ESM.tiff]
